# Acridine Orange/exosomes increase the delivery and the effectiveness of Acridine Orange in human melanoma cells: A new prototype for theranostics of tumors

**DOI:** 10.1080/14756366.2017.1292263

**Published:** 2017-03-06

**Authors:** Elisabetta Iessi, Mariantonia Logozzi, Luana Lugini, Tommaso Azzarito, Cristina Federici, Enrico Pierluigi Spugnini, Davide Mizzoni, Rossella Di Raimo, Daniela F. Angelini, Luca Battistini, Serena Cecchetti, Stefano Fais

**Affiliations:** a Anti-Tumour Drugs Section, Department of Drug Research and Medicines Evaluation, National Institute of Health, Rome, Italy;; b SAFU Department, Regina Elena Cancer Institute, Rome, Italy;; c Neuroimmunology Unit, IRCCS Santa Lucia Foundation, Rome, Italy;; d Department of Cell Biology and Neuroscience, National Institute of Health, Rome, Italy

**Keywords:** Acridine Orange, delivery system, exosome, macrophage, theranostics

## Abstract

Specifically targeted drug delivery systems with low immunogenicity and toxicity are deemed to increase efficacy of cancer chemotherapy. Acridine Orange (AO) is an acidophilic dye with a strong tumoricidal action following excitation with a light source at 466 nm. However, to date the clinical use of AO is limited by the potential side effects elicited by systemic administration. The endogenous nanocarrier exosomes have been recently introduced as a natural delivery system for therapeutic molecules. In this article, we show the outcome of the administration to human melanoma cells of AO charged Exosomes (Exo-AO), in both monolayer and spheroid models. The results showed an extended drug delivery time of Exo-AO to melanoma cells as compared to the free AO, improving the cytotoxicity of AO. This study shows that Exo-AO have a great potential for a real exploitation as a new theranostic approach against tumors based on AO delivered through the exosomes.

## Introduction

Systemic chemotherapy is the most commonly used therapeutic approach against solid tumors, although numerous limitations exist since it is mostly based on pulsatile administration of the maximum tolerated doses (MTDs) of cytotoxic drugs. The long suspension intervals between therapies not only allow patient recovery from various side effects, especially myelosuppression, but also allow, the drug-treated tumors to recover and repopulate as well^1–6^. Unfortunately, many drugs have a very narrow therapeutic index and their clinical use is frequently combined with significant systemic side effects^7–9^. Moreover, delivering drugs to cancer sites at therapeutic concentrations and maintaining adequate drug levels within tumors have proven difficult to be achieved^10–12^. Indeed, systemic chemotherapy faces several limiting factors: (i) drug transport along the blood circulatory system to tissues, (ii) interstitial space, (iii) drug removal by capillaries, (iv) tissue structure and composition with respect to the drug distribution and, probably the most important and (v) the tumor acid extracellular microenvironment^10–14^. As a result, only a fraction of the administered dose reaches tumor cells. Moreover, chemotherapy at the tumor site has to cope with the acidic extracellular microenvironment that neutralizes most of the weak base chemotherapy agents, through H+-mediated protonation, and the alkaline cytoplasm within the tumor cells that neutralizes the same agents with the mechanism of ion trapping^13–18^. However, we had the evidence that using drugs whose metabolism is pH dependent, such as proton pump inhibitors, protonation within the acidic tumor microenvironment led to a full activation of the pro-drug, in turn leading to either tumor chemosensitization^18–20^, or an amazing cytotoxic effects against tumors of different histologies^21–25^, and this was consistent with inhibition of V-ATPase-like proton pumps^26^^,^^27^. These preclinical results supported some clinical trials in either human^28^^,^^29^ or domestic animal^30^^,^^31^ cancer patients, provided the clinical evidence that targeting the acidic tumor microenvironment with drugs that have a preferential delivery for acidic milieu and are fully active, could represent an attractive new approach against cancer^32^. This led also to the setup of an international society that collected virtually all the scientists worldwide investigating the pathophysiological of acidity in human diseases and possible therapeutic approaches to overcome it^33^. Through this international society, we knew the studies of Katsuyuki Kusuzaki (Department of Orthopaedic Surgery, Kyoto Kujo Hospital), on the use of Acridine Orange (AO) in the local treatment of human sarcomas^34^. Acridine Orange is a basic dye synthesized in the nineteenth century that densely accumulates in the acid environment of the lysosomes. Since the tumor extracellular pH (pHe) falls in the acid spectrum due to anaerobic glycolysis, it acts as an attractant to AO^13^. AO has different fluorescence; when AO is illuminated with blue light (450–490 nm), it is visualized as green fluorescence (emission: 515–565 nm), while if the illumination is switched to the green light (546 nm) mode, AO is detected as red fluorescence (emission: 590 nm)^35–37^. AO has the property of being activated by radiation and by specific wavelength (466 nm) (blue light) that results in tumoricidal effect^36–38^. These properties have been in a first time investigated *in vitro* and *in vivo*, showing that AO could be used to detect (upon excitation from a proper light source) tumors, metastases and residual disease after surgical excision^34^^,^^39–41^. The first preclinical investigations prompted clinicians to take advantage of the preferential accumulation of AO in the acid environment of musculoskeletal tumors to detect minimal residual disease, thus allowing limb sparing procedure^42–44^. The same group has more recently described the successful outcome of patients with advanced high grade sarcoma, rhabdomyosarcoma and musculoskeletal sarcoma, treated with surgery and adjuvant therapy with AO excited by radiation and photodynamic therapy^45–48^. The results of these preliminary clinical trials compare favorably with those where standard treatments have been adopted, both in terms of limb functionality preservation (or limb sparing versus amputation) and overall survival. Moreover, the rather small dose of radiation applied to the tumor bed to excite AO was much lower than the standard dose of therapeutic external beam radiation that is currently adopted for these neoplasms, thus avoiding the acute (dermatitis, moist desquamation, delayed would healing) and late (abnormal fibrosis, bone necrosis, radiation induced tumors) side effects associated with radiation therapy^45–49^. The limiting factor of AO is thus its potential systemic toxicity, that has so far confined its application to local therapy, usually by topic administration^34^^,^^38^^,^^50^. There is therefore the need for novel strategies to implement the use of AO in clinical practice and to allow the systemic administration, thus allowing the treatment of distant tumor foci, together with the primary lesions. However, what was challenging in AO was its ability to potentially work as both tracer (being naturally fluorescent) and a cytotoxic drug (following light stimulation). Unfortunately, AO may have problems of delivery by systemic administration and identification of suitable delivery system for AO, in order to increase its effectiveness and reduce potential toxicity, was mandatory.

Exosomes are nano-sized extracellular vesicles (EVs) (30–100 nm in diameter) secreted by virtually all cells of our body, including epithelial and hematopoietic cells, but from cancer cells as well^51^. These nanovesicles are released into the extracellular microenvironment after the fusion of multivesicular bodies with the plasma membrane^51^. They are deeply involved with intercellular communication through the transfer of mRNA, microRNA, receptors and enzymes between cells. Recently, it has been reported that exosomes could be exploited to deliver exogenous RNAs (siRNAs and miRNAs) or chemotherapy agents (doxorubicin)^52^^,^^53^, to tumor xenografts carried by laboratory animals, leading to tumor growth inhibition^54–56^. The use of exosomes and EVs in medical research is impressively increasing year by year, and only recently a network of European researchers published a perspective article that summarized the existing clinical evidence supporting the importance of nano-sized EVs in either diagnosis or therapy of human disease, thus proposing their future use in NanoMedicine^57^.

In the last years, we have been extensively working on exosomes both in terms of characterization and preclinical evaluation showing that these nanovesicles could act as promising instruments for drug delivery and for diagnostic applications^58–69^. Moreover, we had clear evidence that exosome release is pH dependent and that electrostatic gradients between exosomes and a target cells are key in modulating the exosome uptake by the cells^68^. Moreover, we had the evidence that exosomes could deliver fully effective drugs^63^. Lastly, exosomes are acidic vesicles and therefore potential targets for AO, being an acidophilic molecule. Thus, we hypothesized that exosomes could represent the ideal natural nanovector for AO.

The aim of this study was the evaluation of the exosomes to be charged with AO (Exo-AO), the identification of the most efficient exosomal system for the delivery of AO, the quantification of this exosomal cargo, the assessment of the timeframe through which the Exo-AO was released by its carrier and last but not least, the evaluation of the tumoricidal effect of the Exo-AO formulation.

## Methods

### Chemicals and reagents

Acridine Orange powder was purchased from Sigma-Aldrich (Milan, Italy) and resuspended at concentration of 100 µg/ml in phosphate buffer saline (PBS). RPMI 1640 (BE12-702F), antibiotics (DE17-603E), PBS (BE17-512F), trypsin/EDTA (BE17-171E) and fetal bovine serum (FBS) (DE14-701F) were from Lonza (Milan, Italy). Trypan blue was from Alexis Biochemicals (Florence, Italy).

### Cell lines

Metastatic melanoma cell lines Me 30966, supplied by "Istituto Nazionale per lo Studio e la Cura dei Tumori", Milan, Italy, were maintained in RPMI-1640 medium supplemented with 10% fetal calf serum (FCS) and antibiotics, at 37 °C in humidified 5% CO_2_. Human macrophage was obtained after separation of peripheral blood mononuclear cell by Ficoll-Hypaque (Pharmacia, Uppsala, Sweden) density gradient and then by 46% Percoll (Biochrom KG, Berlin, Germany) density gradient of buffy coats from healthy donors. Monocytes were left to differentiate for 1 week at 37 °C in RPMI 1640 plus 20% FBS.

### Exosomes isolation and charging

Exosomes were purified from culture supernatant of macrophages isolated from peripheral blood of healthy donors. The cell culture medium was subjected to differential centrifugation as described in standard protocol for exosomes preparation^70^. Briefly, cell culture medium was centrifuged at 300×*g* (five minutes), 1200×*g* (20 minutes) and 10,000×*g* (30 minutes) to eliminate cells and debris, followed by ultracentrifugation for 1 h and 30 minutes at 100,000×*g* using a Sorvall WX Ultra Series centrifuge in a TH641 rotor (Thermo Scientific, Darmstadt, Germany). The exosome pellets were washed once in a large volume of PBS, centrifuged at 100,000×*g* for 1 h and re-suspended in 50–100 µl of PBS. 100,000×*g* exosome pellet protein recovered were measured by Bradford assay (Bio-Rad, Hercules, CA). Exosomes were used as fresh preparation. After the successful isolation, the obtained exosomes were charged by putting them in contact with a solution of AO at the concentration of 100 µg/ml for 30 minutes at room temperature. After 30 minutes Exo-AO were isolated through centrifugation at 100,000×*g* in a F50L-2461.5 rotor (Thermo Scientific, Darmstadt, Germany) for 1 h.

### Nanoscale flow cytometry analysis of exosomes

Exosomes purified from macrophage cell culture supernatants were diluted in PBS in a final volume of 30 µl. Anti-human CD81 allophycocyanin (APC) conjugated (Beckman Coulter, Brea, CA) and anti-human CD-9 APC-Alexa fluor 750 (Beckman Coulter, Brea, CA) were added to the exosome preparation at optimal pre-tittered concentrations and left for 20 minutes in dark at RT. 500 µl of PBS were added to samples before the acquisition on the CytoFLEX flow cytometer (Beckman Coulter, Brea, CA). The cytometer was calibrated using ApogeeMix beads (Apogee Flow Systems, Middlesex, UK), a mixture of non-fluorescent silica beads and fluorescent (green) latex beads with sizes ranging from 110 nm to 1300 nm. This calibration step enables the determination of the size of EVs. All samples were acquired for the same amount of time in order to obtain an estimate of absolute counts of exosomes comparable between various samples. The analysis of the data was performed with FlowJo software (FlowJo, LLC, Ashland, OR).

### Evaluation of exosomal pH

Exosomal pH was evaluated by nanoscale flow cytometry using the pH-sensitive fluorescent probe BCECF-AM (Molecular Probes, Eugene, OR). Exosome preparations from macrophage cell lines were stained with anti-human CD81, CD9 and incubated at 37 °C for 30 min in PBS containing 20 Amol/l BCECF-AM. The exosomes were then washed in PBS, placed on ice, and analyzed with a CytoFLEX flow cytometer (Beckman Coulter, Brea, CA).

### Cell death determination

Melanoma cells Me 30966 were plated at 4 × 10^4^ cells per well in 12-well plates in 1 ml of buffered RPMI medium. Cells were treated with increasing doses of AO (1, 0.5, 0.25 and 0.10 µg/ml) for 30 minutes, 3 and 6 h. After treatment, samples were washed with PBS and excited with light at 466 nm for 10 s. Then cells were collected by pooling them from the medium (i.e., dead cells) and adherent cells following trypsinization. Cells were then collected (five minutes at 500×*g*), resuspended in PBS/0.4% trypan blue 1:1 (vol/vol). After 10 minutes, cells were analyzed by FACS on a Becton Dickinson FACScalibur using CellQuestPro software (Becton Dickinson System, Franklin Lakes, NJ). For each sample, the total events were acquired in 30 seconds. All experiments were run in triplicate wells and repeated at least twice.

### AO exosomal content quantification by fluorescence

Exosomal content of AO has been quantified using a spectrofluorimeter Perkin-Elmer LS-50B (Waltham, MA). As preliminary step, a calibration curve obtained through the use of increasing concentrations of AO (going from 0.1 to 1 µg/ml) has been performed. In the second step, almost 50 µg of Exo-AO were exposed to fluorescence and their emission was measured. In the third step, we matched the value of emission of the exosomes against those of the calibration curve, obtaining a concentration value. The auto-fluorescence background generated by uncharged exosomes was deducted from the overall fluorescence. All the experiments were carried on using plates for fluorescence using light at the wavelength of 466 ± 5 nm in excitation and 525 ± 5 nm filter in emission. The experiment has been repeated for six times in duplicate.

### Melanoma spheroids formation and confocal laser scanning microscopy

To allow spheroid formation, 1 × 10^4^ cells/ml of melanoma Me 30966 cells were cultured in 96-well plate (Ultra Low Attachment, Costar, Paris, France) in complete cell culture medium until 72 h at 37 °C and 5% CO_2_ in continuous rotation. Free AO or Exo-AO derived from macrophages (M ϕ Exo-AO) were then added for 10′, 6 h, 24 h or 48 h. After this time, point cells were washed, fixed with 3% paraformaldehyde (PFA) for 30′ at room temperature, seeded in coverslips and then mounted on the microscope slide by using Vectashield^®^ mounting medium (Vector Laboratories Inc., Burlingame, CA). CLSM observations were performed on a Leica TCS SP2 AOBS apparatus (Leica Microsystems, Wetzlar, Germany), using excitation spectral laser lines at 488 and 546 nm, and using the confocal software (Leica, Wetzlar, Germany) and Photoshop CS2 (Adobe Systems, San Jose, CA). Signals from different fluorescent probes were taken in sequential scanning mode, several fields were analyzed for each labeling condition, and representative results are shown. Images were obtained by Z-projection of 18–20 optical sections taken from the bottom to the edge of the spheroids.

### Melanoma spheroids and cell death determination

To allow spheroid formation, as above described, free AO (5 µg/ml) or M ϕ Exo-AO (5 µg/ml) were then added for 6 h. Then melanoma spheres were washed with PBS and exposed to blue light (466 nm) for 36 s, then plated again in complete cell culture medium for 24 h. After this period, five spheroids were pooled and cell death was measured with acid phosphatase assay. Briefly, one tablet of p-nitrophenyl phosphate disodium hexahydrate was dissolved in 10 ml of sodium acetate buffer (0.1 M of sodium acetate, 0.1% of Triton X100) at pH 5.0. Then 100 µl of substrate (p-nitrophenyl phosphate) were added to the plate and the spheroids were incubated at 37 °C for 1 h. To arrest the reaction, 10 µl of sodium hydroxide 1 M were added and fluorescence was measured with a spectrophotometer ELISA reader (Elx800). Absorbance of p-nitrophenol at 405 nm is directly proportional to the number of viable cells. All experiments were run in triplicate and repeated at least twice.

### Fluorescence microscopy observation

Melanoma cells Me 30966 were plated at 4 × 10^4^ cells per well in 12-well plate in 1 ml of buffered RPMI medium. The day after, cells were treated with M ϕ Exo-AO at concentration 1 µg/ml for six hours. After treatment, cells were washed with PBS and excited to light at 466 nm for 10 s. Then the plate was placed on the stage of an inverted Zeiss Axiovert 135 TV fluorescence microscope (Carl Zeiss, Oberkochen, Germany) equipped with a digital camera. Then, cells were illuminated with blue light, which was produced by a mercury short ARC photo optic lamp HBO 103 W passing through an excitation filter (450–490 nm) and a heat-protecting filter. The light intensity was maintained at 100% of the maximum intensity by an Attoarc device (Carl Zeiss, Oberkochen, Germany). The fluorescence was viewed as green through an emission filter (515–565 nm). The photomicrographs were taken by digital camera (AxioCamMRc 5, Carl Zeiss, Oberkochen, Germany) driven by software AxioVision 4.2 (Carl Zeiss, Oberkochen, Germany).

### Statistical analysis

Results are expressed as the means ± SD using paired Student's *t* tests and one-way ANOVA. A Bonferroni *t*-test was used to determine group differences. *p < 0.05 was regarded as significant.

## Results

### Macrophage-derived EVs can be successfully charged with AO

**Figure 1.**
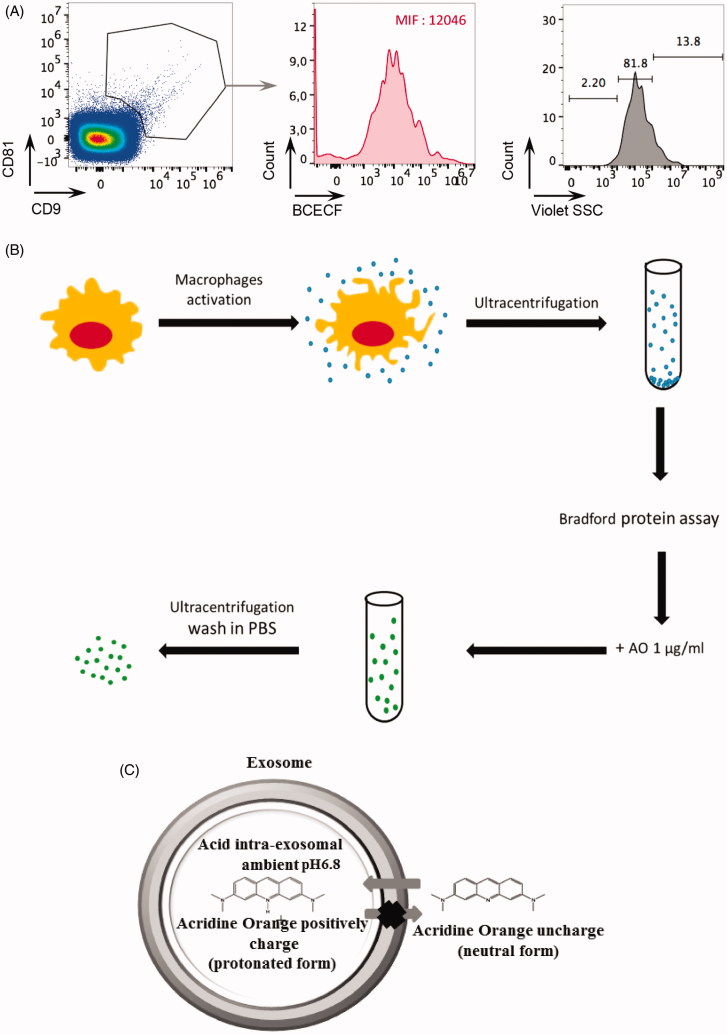
(A) The exosome preparation derived from human macrophage supernatants was stained with anti-CD9 and anti-CD81 antibodies and analyzed by nanoscale-flow cytometry. The double positive events were then analyzed for their size. Relative percentages are shown on plots. CD9+ CD81+ microvesicles of size less than 110 nm were also analyzed by flow cytometer for the acidic content using the BCECF dye. Within total CD81+ CD9+ exosomes population, the median intensity fluorescence (MIF) of BCECF was evaluated to measure the intravesical pH. High MIF value corresponds to high pH. Data are expressed as means ± SD of three independent experiments. (B) Proposed experimental protocol for AO charging on macrophage exosome preparation. (C) Proposed mechanism of AO charged exosome retention.

Extracellular vesicles were purified from human macrophage cell culture supernatants by repeated rounds of ultracentrifugation, as described in Théry et al.^70^. Purified EVs were first characterized by identification of standard exosomal markers with either nanoscale-flow cytometry (Figure 1(A)) or western blot analysis (data not shown). The double positive events were counted and analyzed for size (Figure 1(A)). We found that EV preparations are enriched with EVs of size less than 110 nm (81.8%) (Figure 1(A)). These macrophage-derived EVs (M ϕ EVs) were then exposed to 100 µg/ml of AO (see Figure 1(B)), and the exosomal content of AO was quantified using a spectrofluorimeter. The data strongly suggested that AO diffused within the macrophage derived exosomes. In fact, M ϕ exosomes carried 0.036 µg of AO per 1 µg of exosomal protein. AO can bind DNA or RNA by intercalation. It is well known that exosomes contain various kinds of RNA or DNA. Therefore, to exclude the binding of AO to DNA or RNA potentially contained into M ϕ EVs, CD9/CD81 double positive M ϕ EVs with size less than 110 nm were analyzed by nanoscale-flow cytometry for the detection of their acidic content with the BCECF ester probe, that measures pH between 6.8 and 6.5. Interestingly, we found a median fluorescence of 12,046, strongly suggesting not only that M ϕ EVs are acidic in their internal content but also that the accumulation of AO into M ϕ EVs is favored by an acidophilic binding. These results provided the first evidence that (a) EVs could be successfully charged with AO (Figure 1(C)); (b) simple diffusion of a weak base drug, such as AO, is an effective tool to charge EVs with a drug, simply exploiting their constitutive acidity (Figure 1(C)).

### Identification of the most efficient delivery system

**Figure 2.**
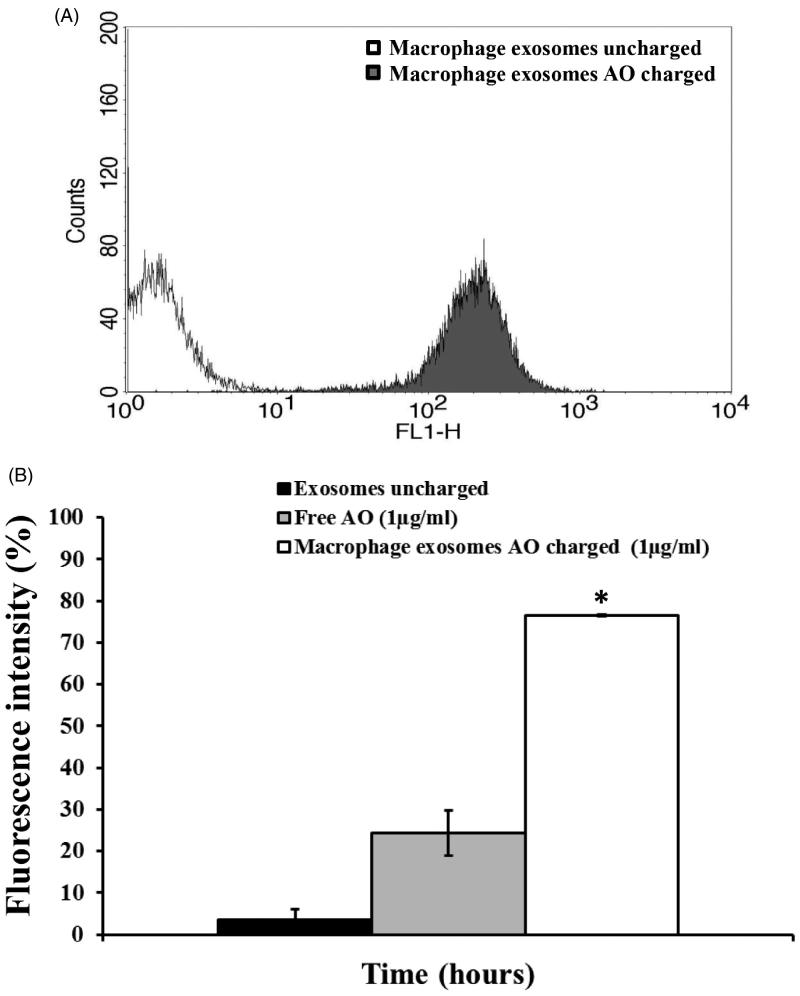
(A) Cytofluorimetry assessment of the potential uptake of 25 μg M ϕ Exo-AO on human melanoma Me 30966 treated for 10 minutes. (B) Cytofluorimetric evaluation of the potential release of M ϕ Exo-AO versus free AO on human melanoma Me 30966 treated for 6 h. Columns, mean percentages of fluorescence intensity of two independent experiments run in triplicate; bars indicate SD. *p < 0.05.

In the first experiment, when using 25 µg of exosomal protein, we have detected a huge emission of fluorescence by melanoma cells treated for six hours with M ϕ Exo-AO (Figure 2(A)), indicating that these exosomes are able to release very efficiently their AO cargo. In the second set of experiments, we compared the effect, over a prolonged period of time (6 h), induced by 1 µg/ml of AO charged within the M ϕ Exo. These data were matched against the results obtained exposing melanoma Me 30966 cells to free AO. In these experiments, it has been clearly demonstrated that Exo-AO were still significantly present and detectable within its cellular targets after six hours from the treatment, while free AO was present in much lower concentration in its targets as shown by fluorescence intensity (Figure 2(B)).

### Macrophage derived exosomes carrying AO result in greater antitumor efficacy against melanoma cells than free AO

In medical oncology, free AO is the current clinical standard and the only safe way to use this drug in patients^44^. On the other hand, macrophage derived exosomes can be safely used in a therapeutic setting, due to the potential transforming action of the tumor derived exosomes^62^.

**Figure 3.**
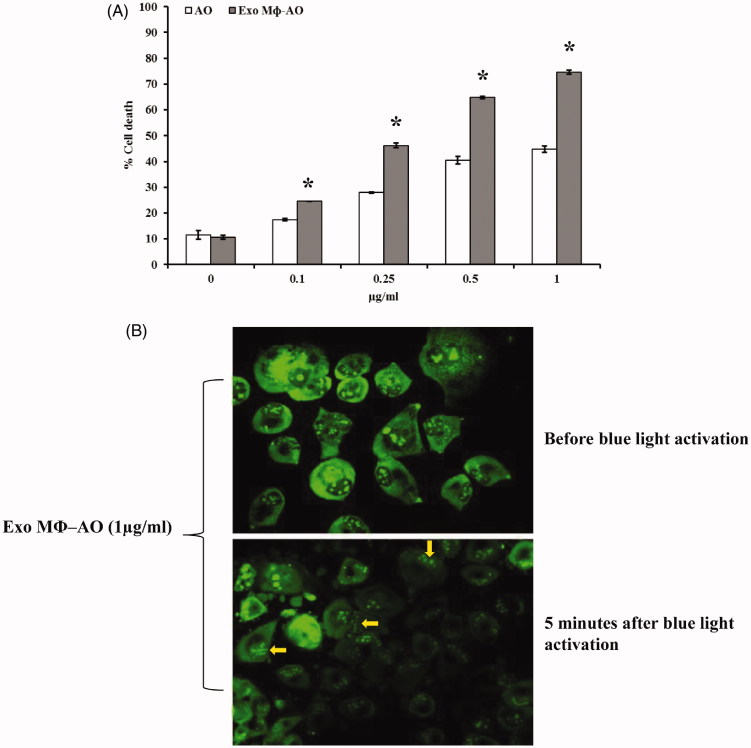
(A) Cytotoxic effect of M ϕ Exo-AO compared to free AO against melanoma cell monolayer by cytofluorimetry assessment. Columns, mean percentages of cell death of two independent experiments run in triplicate; bars indicate SD. *p < 0.05. (B) Fluorescence microscopy showing the formation of membrane "blebs" in melanoma cells treated with M ϕ Exo-AO (1 μg/ml) after five minutes of exposition to blue light.

The cytotoxic effect of AO was compared to that of M ϕ Exo-AO in monolayer culture (2D) condition of human melanoma cells (Me 30966). The effect was evaluated at 30 minutes, 3 h and 6 h after the exposure and the outcome was in accordance with the uptake data. The cytotoxic effect after a cell exposure to either free AO or M ϕ Exo-AO, and its activation by blue light (10 seconds) at the wavelength of 466 nm, was comparable at 30 minutes and 3 h (data not shown). Finally, in accordance with the uptake-retention pattern M ϕ Exo-AO showed, at six hours after treatment, a greater cytotoxic effect than free AO (*p* < 0.05), while more evident at the highest concentrations (Figure 3(A)). To exclude that exosomes derived from macrophages have itself any anti-cancer effect, we included in the experiments co-cultivation of melanoma cells with M ϕ-derived EVs without the addition of drugs and we never observed any cytotoxic effect against melanoma cells (data not shown). These findings were substantiated by another set of experiments that showed the preservation of the radical forming property by M ϕ Exo-AO. In fact, fluorescence microscopy confirmed the presence of the bleb forming phenomenon (arrows) in the cells treated with 1 µg/ml of M ϕ Exo-AO (Figure 3(B)) and then excited as above described.

### MΦ Exo-AO result in longer retention and increased cytotoxicity than free AO in 3D culture condition

**Figure 4.**
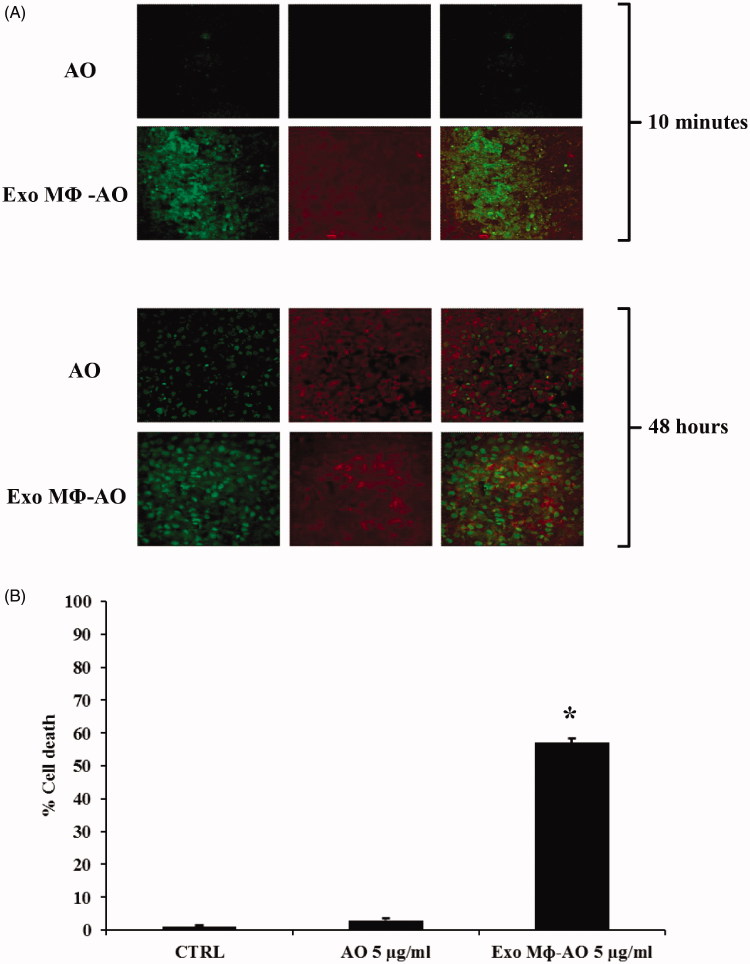
(A) Confocal laser scanning microscopic assessment of release and retention of M ϕ Exo-AO versus free AO in melanoma spheroids at 10 minutes and 48 h of incubation. (B) Cytotoxicity induced in melanoma spheroids by M ϕ Exo-AO versus free AO. Columns, mean percentages of cell death of two independent experiments run in triplicate; bars indicate SD. *p < 0.05.

In this set of experiments, it has been shown that in the three-dimensional (3D) model of human melanoma, free AO was slowly uptaken by target cells (Me 30966). On the other hand, M ϕ Exo-AO was immediately and homogenously uptaken by target cells within 10 minutes of exposure (Figure 4(A)). Trying to figure out the possible behavior of free AO and M ϕ Exo-AO within solid tumors, we evaluated the uptake and release of both agents over 48 h. We ascertained that after 6 h all forms of AO, either free or delivered by M ϕ exosomes, reached an even distribution within the melanoma spheroids preferentially localized within the acid cytoplasmic compartments as suggested by the green fluorescence observed by confocal microscopy. At 24 h checkpoint, the fluorescence of spheroids treated with M ϕ Exo-AO was higher than those treated with free AO (data not shown). However, at the 48 h final checkpoint, the residual fluorescence was much lower in the spheroids treated with free AO than M ϕ Exo-AO (Figure 4(B)).

In the same set of experiments, we evaluated the level of cytotoxicity against the 3D melanomas, again comparing free AO to M ϕ Exo-AO. We thus measured the percentage of spheroids cell death, following activation by blue light (10 seconds) at the wavelength of 466 nm. The results were obtained by means of spectrophotometric analysis. This experiment showed that free AO induced a barely detectable mortality (<5%), quite similar to that of control spheroids, while M ϕ Exo-AO accounted for a 57% mortality (*p* < 0.01) (Figure 4(C)). This set of experiments thus definitively demonstrated that AO delivered by macrophage-derived exosomes (M ϕ Exo-AO) had a straightforward anti-tumor effect (as simulated by 3D culture conditions) once exited by the blue light, as compared to the free AO.

## Discussion

Melanoma is a well-known chemotherapy refractory tumor where survival is dependent on early tumor detection and surgical removal, with Breslow’s depth of the neoplasm and the status of the sentinel node(s) carrying the highest prognostic value for the patients^71–74^.

Unfortunately, to date, there are no therapeutic procedures that can counter the metastatic spread of this tumor and patients with metastatic melanoma have a dismal prognosis^75–78^. Considering that melanoma is a highly acidic neoplasm^22^ and that tumor metastases, being biologically more aggressive, are also more acidic than primary tumors^20^^,^^32^^,^^79^, we considered AO a potential useful addition to the armory available to clinicians. To overcome the intrinsic toxicity of AO, when used systemically^80^^,^^81^, we devised a novel intriguing strategy involving the use of exosomes as carriers. In our study, we show for the first time that exosomes could be successfully charged with AO, preserving the mechanism of action of the drug and without changes in the chemical–physical properties of the exosomes. More importantly, the exosome delivery system showed to actually enhance the tumoricidal effect of AO, by increasing the exposure time of the biological targets. In fact, M ϕ Exo-AO was taken much more rapidly within melanoma cells and was retained for a longer time than free AO, at the same time achieving a significant tumoricidal effectiveness, in the 3D condition, such as spheroids. This carries the potential for clinical application to cancer patients, exploiting the inherent tumor acidity against the tumor itself by delivering and releasing larger amounts of antitumor agents, at the same time sparing the normal tissues. Another advantage that has been already exploited at a local level, is the possibility to selectively activate this agent through the exposure to light having the appropriate wavelength, thus adding another safety to the system.

The intracytoplasmic release potential evidenced by Exo-AO cannot be entirely explained by the concept of simple drug diffusion, since the charging continued beyond the reach of the expected equilibrium point. Therefore it is conceivable that these different drug kinetics involve other factors responsible for the chemical–physical properties displayed by the Exo-AO. Possible mechanisms involved in this phenomenon could be: lipid–lipid interaction, electrostatic interaction or transmembrane active transport. In fact, we have previously shown that exosomes may interact with target cells undergoing fusion with the plasma membrane of the cells, and this is highly dependent on the electrostatic charges of the interacting membranes^68^. It is to emphasize that AO actually represents an ideal model of a molecule that at the same time is a tracer (being fluorescent) and a highly cytotoxic molecule following the blue light stimulation^34^. In fact, the use of AO against malignant tumors was included between the so called "photodynamic therapies". This is clearly an advantage inasmuch as it allows to induce tumor cytotoxicity only when the drug (i.e. AO) is delivered to the tumor site through external photostimulation. Thus, our study not only provides the evidence of an entirely new mechanism of drug delivery to the tumor site through an autologous natural delivery system (i.e. exosomes), but introduces the use of an external control of the therapeutic activation of the drug through photostimulation.

We consider the results of our study are of paramount importance for the future development of the "Theranostics" approach in Nanomedicine. Of course our group is programing additional investigations to further characterize this successful combination and to test its efficacy in different tumor histotypes.
